# Targeting S100A9 Reduces Neutrophil Recruitment, Inflammation and Lung Damage in Abdominal Sepsis

**DOI:** 10.3390/ijms222312923

**Published:** 2021-11-29

**Authors:** Zhiyi Ding, Feifei Du, Richard Garland Averitt V, Gabriel Jakobsson, Carl-Fredrik Rönnow, Milladur Rahman, Alexandru Schiopu, Henrik Thorlacius

**Affiliations:** 1 Department of Clinical Sciences, Malmö, Section for Surgery, Lund University, 21428 Malmö, Sweden; zhiyi.ding.3635@med.lu.se (Z.D.); feifei.du.0320@med.lu.se (F.D.); quinn@raveritt.com (R.G.A.V.); carl-fredrik.ronnow@med.lu.se (C.-F.R.); milladur.rahman@med.lu.se (M.R.); 2 Department of Clinical Sciences, Malmö, Lund University, 21428 Malmö, Sweden; gabriel.jakobsson@med.lu.se (G.J.); alexandru.schiopu@med.lu.se (A.S.); 3 Department of Internal Medicine, Skåne University Hospital, 22185 Lund, Sweden

**Keywords:** sepsis, infection, inflammation, leukocyte, lung

## Abstract

S100A9, a pro-inflammatory alarmin, is up-regulated in inflamed tissues. However, the role of S100A9 in regulating neutrophil activation, inflammation and lung damage in sepsis is not known. Herein, we hypothesized that blocking S100A9 function may attenuate neutrophil recruitment in septic lung injury. Male C57BL/6 mice were pretreated with the S100A9 inhibitor ABR-238901 (10 mg/kg), prior to cercal ligation and puncture (CLP). Bronchoalveolar lavage fluid (BALF) and lung tissue were harvested for analysis of neutrophil infiltration as well as edema and CXC chemokine production. Blood was collected for analysis of membrane-activated complex-1 (Mac-1) expression on neutrophils as well as CXC chemokines and IL-6 in plasma. Induction of CLP markedly increased plasma levels of S100A9. ABR-238901 decreased CLP-induced neutrophil infiltration and edema formation in the lung. In addition, inhibition of S100A9 decreased the CLP-induced up-regulation of Mac-1 on neutrophils. Administration of ABR-238901 also inhibited the CLP-induced increase of CXCL-1, CXCL-2 and IL-6 in plasma and lungs. Our results suggest that S100A9 promotes neutrophil activation and pulmonary accumulation in sepsis. Targeting S100A9 function decreased formation of CXC chemokines in circulation and lungs and attenuated sepsis-induced lung damage. These novel findings suggest that S100A9 plays an important pro-inflammatory role in sepsis and could be a useful target to protect against the excessive inflammation and lung damage associated with the disease.

## 1. Introduction

Abdominal sepsis is a fatal condition triggered by systemic spread of microbial pathogens, resulting in wide-spread stimulation of the innate immune system. Neutrophils are a key component in the innate immune system and excessive activation of neutrophils is known to cause organ dysfunction in sepsis [1,2]. The lung is the most vulnerable organ in abdominal sepsis and the most feared complication of septic lung damage is compromised exchange of oxygen and nutrients in the pulmonary microcirculation. Pulmonary infiltration of neutrophils is known to be a rate-limiting step in sepsis-induced lung injury [3,4]. Targeting specific adhesion molecules has been shown to reduce not only neutrophil recruitment but also tissue damage in septic lungs. Extravascular accumulation of neutrophils is coordinated by secretion of CXC chemokines, including CXCL1 and CXCL2 [5,6]. 

Neutrophils cause tissue injury via secretion of reactive oxygen species, proteolytic enzymes and expulsion of neutrophil extracellular traps (NETs) [7,8,9,10]. In addition, neutrophils contain calprotectin, a S100A8/A9 heterodimer protein with numerous pro-inflammatory effects [11,12]. S100A9 constitute up to 45% of all cytoplasmic proteins in neutrophils [13,14]. The protein is also expressed in monocytes and dendritic cells, but at much lower levels [15,16]. S100A9 is up-regulated in various models of inflammation and infection [17,18,19]. S100A9 is an alarmin which serves to warn the host of imminent danger [20]. Increased formation of S100A9 is observed in patients with sepsis [21] and severe COVID-19 infection [22]. Notably, convincing data have shown that calprotectin can stimulate neutrophil migration and accumulation at sites of inflammation [23,24]. However, the role of S100A9 in abdominal sepsis is elusive. 

Based on the considerations above, we hypothesized that targeting S100A9 might reduce secretion of pro-inflammatory mediators, accumulation of neutrophils and tissue damage in sepsis-induced lung damage. 

## 2. Results

### 2.1. Septic Levels of S100A9

**Figure 1:**
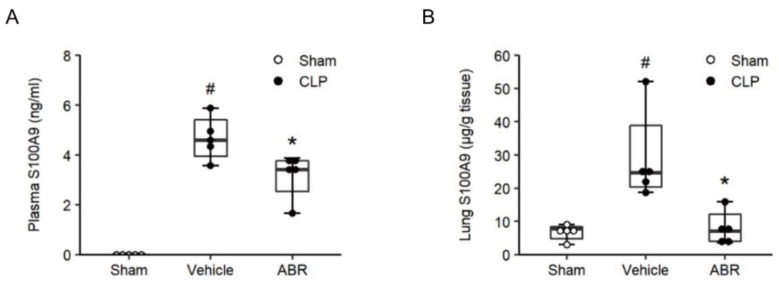
Levels of S100A9 in plasma and lung. Mice were pre-treated with intraperitoneal injections of the vehicle or ABR-238901 (10 mg/kg) 1 h before Sham or CLP operation. Levels of S100A9 in (**A**) plasma and (**B**) lung were determined 24 h after CLP induction. Data are presented as box plots with median (25–75 percentile) and the whiskers extend from the minimum to the maximum levels and dots represent sample values. *^#^ p* < 0.05 vs. Sham, and * p < 0.05 vs. Vehicle, *n* = 5.

Plasma and lung levels of S100A9 were undetectable or low in sham animals (Figure 1A,B). Induction of CLP increased plasma levels of S100A9 to 4.6 ng/mL (Figure 1A). Moreover, CLP elevated lung levels of S100A9 to 24.7 μg/g tissue, which corresponds to a 3.6-fold increase compared to sham animals (Figure 1B). Administration of ABR-238901 decreased CLP-induced levels of S100A9 in plasma and lungs by 26% and 95%, respectively (Figure 1A,B).

### 2.2. Neutrophil Recruitment and Mac-1 Expression

**Figure 2:**
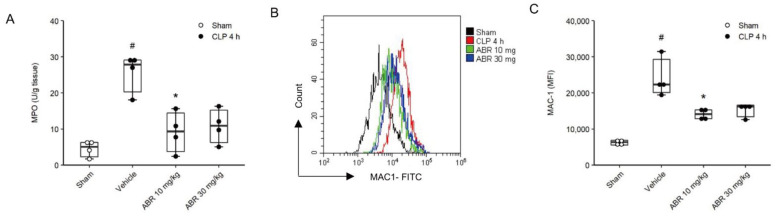
S100A9 regulates neutrophil recruitment. Mice were pre-treated with intraperitoneal injections of vehicle or ABR-238901 (10 mg/kg and 30 mg/kg) 1 h before CLP. (**A**) Lung MPO was determined 4 h after CLP. (**B**) Blood was collected 4 h after CLP and Mac-1 expression (MFI) on neutrophils was determined by flow cytometry. (**C**) Aggregate data on Mac-1 expression. Statistical comparisons were performed using non-parametrical tests (Kruskal–Wallis on ranks followed by Dunn’s multiple comparisons). Data are presented as box plots with median (25–75 percentile) and the whiskers extend from the minimum to the maximum levels and dots represent sample values. *^#^ p* < 0.05 vs. Sham, and * p < 0.05 vs. Vehicle, *n* = 4.

**Figure 3:**
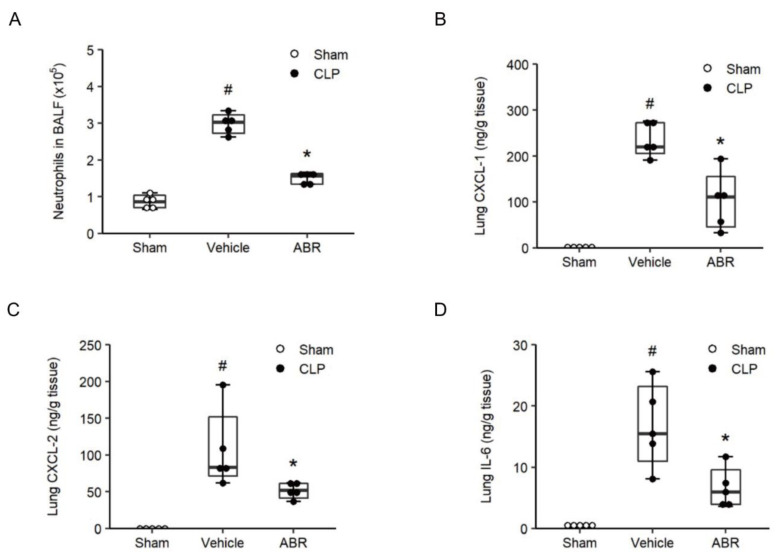
S100A9 regulates neutrophil recruitment. (**A**) BALF neutrophils were quantified in Bronchoalveolar lavage fluid (BALF) collected 24 h after CLP induction. Levels of (**B**) CXCL-1, (**C**) CXCL-2 and (**D**) IL-6 in the lungs were determined 24 h after CLP induction. Data are presented as box plots with median (25–75 percentile) and the whiskers extend from the minimum to the maximum levels and dots represent sample values. *^#^ p* < 0.05 vs. Sham, and * p < 0.05 vs. Vehicle, *n* = 5.

Four hours after CLP, lung levels of MPO, a marker of neutrophil activation, increased by more than 5.6-fold to 27.9 U/g tissue (Figure 2A, *p* < 0.05 vs. sham, *n* = 4). Administration of 10 and 30 mg/kg of ABR-238901 decreased MPO levels by 81% and 74%, respectively (Figure 2A, *p* < 0.05 vs. Vehicle + CLP, *n* = 4) in septic mice. CLP increased Mac-1 expression on neutrophils at 4 h compared with sham-operated animals (Figure 2B,C, *p* < 0.05 vs. sham, *n* = 4). Sepsis-induced Mac-1 up-regulation was significantly attenuated by treatment with 10 or 30 mg/kg of ABR-238901 (Figure 2B,C, *p* < 0.05 vs. sham, *n* = 4). Administration of 10 and 30 mg/kg ABR-238901 decreased MFI values of Mac-1 on neutrophils from 22,330 (20,096–29,286) to 14,087 (12,834–15,323) and 16,193 (13,415–16,581), respectively, in CLP mice (Figure 2C, *p* < 0.05 vs. PBS + CLP, *n* = 4). Thus, 10 and 30 mg/kg of ABR-238901 exerted similar anti-inflammatory effects on MPO levels in the lungs and Mac-1 expression on neutrophils in CLP animals and therefore 10 mg/kg of ABR-238901 was used for the remaining experiments. Cell analysis of BALF revealed a clear increase in the number of neutrophils, which increased by 3.5-fold 24 h after CLP (Figure 3A, *p* < 0.05 vs. sham, *n* = 5). Treatment with ABR-238901 (10 mg/kg) decreased pulmonary neutrophils from 3.0 (2.7–3.2) × 10^5^ down to 1.6 (1.3–1.6) × 10^5^, corresponding to a 68% reduction (Figure 3A, *p* < 0.05 vs. Vehicle + CLP, *n* = 5).

### 2.3. CXC Chemokines and IL-6 in the Lung

Pulmonary levels of CXC chemokines in control mice were low (Figure 3B,C, *n* = 5). In contrast, CLP enhanced lung levels of CXCL1 and CXCL2 from 1.1 (0.4–2.0) ng/g and 0 ng/g up to 219.8 (205.2–272.8) ng/g and 83.1 (71.6–152.1) ng/g tissue, respectively (Figure 3B,C, *p* < 0.05 vs. Sham, *n* = 5). Treatment with ABR-238901 (10 mg/kg) reduced CLP-induced generation of CXCL1 and CXCL2 down to 110.8 (44.9–155.5) ng/g and 51.8 (41.1–61.4) ng/g lung tissue, respectively (Figure 3B,C, *p* < 0.05 vs. PBS + CLP, *n* = 5). Thus, ABR-238901 decreased CXCL1 and CXCL2 secretion in the lungs by more than 50% and 38% in septic mice. In addition, administration of ABR-238901 decreased CLP-induced formation of IL-6 by 63% in the lungs (Figure 3D, *p* < 0.05 vs. PBS + CLP, *n* = 5).

### 2.4. Lung Edema and Tissue Injury

**Figure 4:**
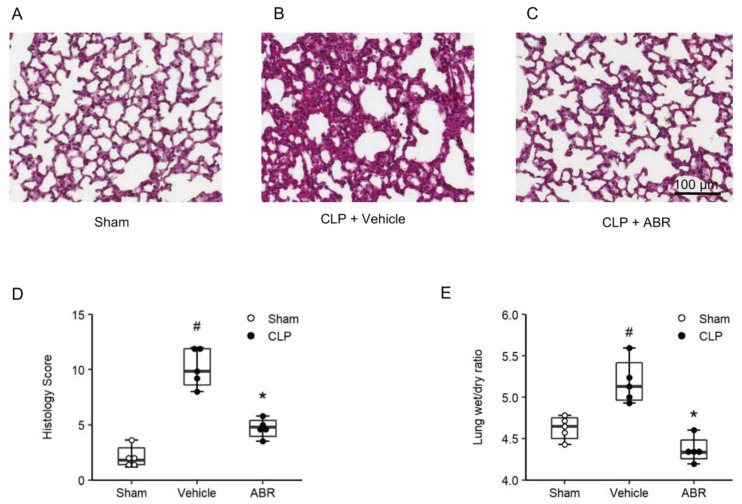
S100A9 promotes septic lung damage. Representative images of (**A**) Sham mice and CLP mice pre-treated with (**B**) vehicle or (**C**) 10 mg/kg of ABR-238901 for 1 h before CLP operation. (**D**) Lung injury score was evaluated by using a predefined scoring system in a double-blinded manner, as described in the method sections. Lung weight wet/dry ratio was used to determine edema formation (**E**). Data are presented as box plots with median (25–75 percentile) and the whiskers extend from the minimum to the maximum levels and dots represent sample values. *^#^ p* < 0.05 vs. Sham, and * p < 0.05 vs. Vehicle, *n* = 5. Scale bars = 100 µm.

Examination of H&E-stained cross sections of the lungs from sham mice exhibited normal pulmonary microarchitecture (Figure 4A), whereas CLP mice demonstrated severe destruction of the lung tissue architecture with interstitial edema, capillary congestion, necrosis and massive infiltration of neutrophils (Figure 4B). Administration of ABR-238901 reduced sepsis-provoked tissue destruction and inflammation (Figure 4C). Quantification of the histological damage revealed that CLP significantly increased the lung injury score (Figure 4D, *p* < 0.05 vs. sham, *n* = 5). Notably, treatment with ABR-238901 reduced the lung injury score in CLP animals by 63% (Figure 4E, *p* < 0.05 vs. PBS + CLP, *n* = 5). Pulmonary edema formation greatly increased in animals subjected to CLP. Thus, we found that lung wet/dry ratio increased by 10%, i.e., from 4.6 (4.5–4.7) to 5.1 (5.0–5.4) in septic animals (Figure 4E, *p* < 0.05 vs. sham, *n* = 5). Administration of 10 mg/kg ABR-238901 reduced decreased wet/dry ratio down to 4.3 (4.2–4.5), in CLP mice, corresponding to an edema reduction of more than 99% (Figure 4E, *p* < 0.05 vs. PBS + CLP, *n* = 5).

### 2.5. Systemic Inflammation

**Figure 5:**
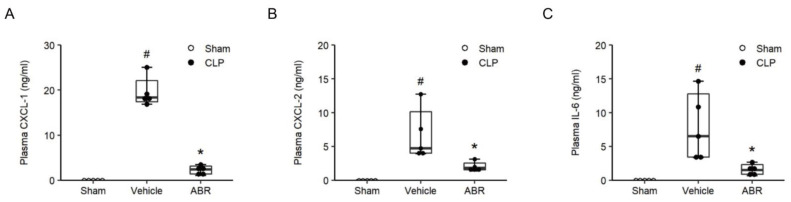
S100A9 regulates systemic inflammation. Mice were pre-treated with intraperitoneal injections of the vehicle or ABR-238901 (10 mg/kg) 1 h before Sham or CLP operation. Levels of (**A**) CXCL-1, (**B**) CXCL-2 and (**C**) IL-6 in plasma were determined 24 h after CLP induction. Data are presented as box plots with median (25–75 percentile) and the whiskers extend from the minimum to the maximum levels and dots represent sample values. *^#^ p* < 0.05 vs. Sham, and * p < 0.05 vs. Vehicle, *n* = 5.

CLP triggered increases of plasma levels of CXCL1 by 18.3-fold, CXCL2 by 4.7-fold and IL-6 by 6.5-fold (Figure 5A–C, *p* < 0.05 vs. sham, *n* = 5). Treatment with 10 mg/kg of ABR-238901 reduced plasma levels of IL-6, CXCL1 and CXCL2 by more than 77%, 87% and 61%, respectively in septic animals (Figure 5A–C, *p* < 0.05 vs. PBS + CLP, *n* = 5). As part of a systemic inflammatory response in sepsis, the number of circulating leukocytes decreases. Indeed, it was found that CLP caused leukocytopenia (Table 1) and that administration of ABR-238901 antagonized CLP-induced leukocytopenia in septic mice (Table 1).

### 2.6. Late Treatment with ABR-238901

In separate experiments, ABR-238901 (10 mg/kg) was administered after CLP induction. However, late administration of ABR-238901 had no effect on neutrophil recruitment, edema and CXC chemokines formation in the lungs of CLP animals (Figures S1 and S2).

## 3. Discussion

Management of patients with sepsis poses a huge challenge to clinicians. Therapy is largely limited to supportive care and novel ways to ameliorate pathological inflammation in abdominal sepsis. This investigation demonstrates that targeting S100A9 reduces septic lung damage. Blocking S100A9 decreased neutrophil activation and pulmonary infiltration and attenuated lung damage in abdominal sepsis. Our findings suggest that S100A9 regulates lung accumulation of neutrophils via both Mac-1 up-regulation on neutrophils and induction of CXC chemokines in the lungs. Thus, this study suggest that S100A9 plays an important role in abdominal sepsis and that targeting S100A9 may be useful to protect against septic lung injury.

S100A9 is best known as a dimerization partner of S100A8, forming calprotectin, which is used as an effective marker of gut inflammation in patients with Crohn’s disease and ulcerative colitis [25,26]. Nonetheless, accumulating data suggest that S100A9 exerts a functional role in several inflammatory conditions, including arthritis [27], myocardial infarction [17,28], endotoxin-induced lung injury [29] and acute pancreatitis [30]. In the present study, it was observed that induction of abdominal sepsis greatly enhanced plasma and lung levels of S100A9. This finding is in line with observation in patients with sepsis and COVID-19 disease [21,22] and supports the notion of S100A9 as an active mediator in severe infections. Notably, our data demonstrate that treatment with ABR-238901, a specific S100A9 inhibitor, markedly decreased CLP-induced elevations of S100A9 in plasma and lungs, also attenuated systemic neutrophil activation, pulmonary chemokines and edema formation as well as tissue damage, suggesting that S100A9 plays an important role in regulating inflammation and lung damage associated with abdominal sepsis. These findings are in line with a previous study, which shows that inhibition of S100A9 inhibits endotoxin-induced lung injury [29]. However, it should be mentioned that another study reported that administration of S100A9 reduced endotoxin-induced lung injury [31]. The reasons for this discrepancy in the endotoxin-based model of lung injury is not known but could be related to different experimental protocols. Interestingly, our present findings extend a previous study demonstrating that mice lacking S100A9 have improved survival and decreased liver damage, in a model based on intraperitoneal injection of Escherichia coli [32]. Together, these results support the concept that targeting S100A9 might be of beneficial value in lung damage triggered by systemic spread of bacteria. Although late treatment with ABR-238901 did not reduce CLP-induced lung inflammation and damage, future studies should evaluate the role of late inhibition of S100A9 in septic animals treated with antibiotics. Nonetheless, several studies have established neutrophil infiltration as a key component in the pathophysiology of septic lung injury [33,34]. In the present study, we observed that administration of ABR-238901 decreased lung levels of MPO by 74% in abdominal sepsis. Moreover, our results reveal that ABR-238901 reduces sepsis-provoked pulmonary neutrophilia by 68%. Together, these findings suggest that S100A9 is a key regulator of neutrophil trafficking in septic lung injury. 

Numerous studies have shown that neutrophil recruitment into the lungs is a multistep process involving microvascular trapping and active adhesion on the endothelium, followed by transendothelial extravasation [35,36]. Leukocyte–endothelial cell interactions are supported by specific adhesion molecules [37]. For example, convincing results have documented that P-selectin glycoprotein ligand-1 (PSGL-1), LFA-1 and Mac-1, on neutrophils, mediate alveolar accumulation in septic lung injury [3,38]. Previous data has also shown that S100A9 triggers Mac-1-dependent neutrophil adhesion to fibrinogen in vitro [39]. We therefore asked whether ABR-238901 could regulate Mac-1 expression on neutrophils in sepsis. We observed that CLP caused a clear increase in Mac-1 expression on circulating neutrophils. Treatment with ABR-238901 significantly decreased neutrophil up-regulation of Mac-1 in septic animals, suggesting that S100A9 indeed regulates Mac-1 expression on neutrophils in abdominal sepsis. Secreted chemokines regulate accumulation of leukocytes at extravascular sites [40,41]. CXC chemokines CXCL1 and CXCL2 are especially effective in attracting neutrophils [42,43]. Notably, it has been documented that S100A9 can activate and promote secretion of CXC chemokines from macrophages [44] and epithelial cells [45,46]. In the present study, we found that ABR-238901 attenuated sepsis-associated CXCL1 and CXCL2 formation by more than 50% and 38% in the lung. A previous study showed that blocking CXCR2, the main receptor of CXCL1 and CXCL2, effectively decreased alveolar accumulation of neutrophils in the CLP model of sepsis [4], demonstrating that CXC chemokines are important regulators of pulmonary infiltration of neutrophils in septic lung injury. We observed that treatment with ABR-238901 greatly decreased lung generation of CXCL1 and CXCL2, suggesting that S100A9 regulates CXC chemokine formation in the inflamed lung. Thus, inhibition of S100A9 reduces both Mac-1 up-regulation on neutrophils and CXC chemokine formation in tissues, which helps to explain part of the inhibitory impact of ABR-238901 on neutrophil recruitment in septic lung injury. It should be mentioned that S100A9 could also regulate other aspects of leukocyte recruitment and further investigation is required to explore the exact role of S100A9 and other alarmins in sepsis and lung injury.

Leukocytopenia is a key hallmark of systemic inflammation [33]. Herein, we observed that ABR-238901 antagonized CLP-induced leukocytopenia, indicating that S100A9 also controls systemic inflammation in sepsis. This notion is also supported by our finding demonstrating that ABR-238901 reduced plasma levels of IL-6, CXCL1 and CXCL2 in septic mice.

Taken together, our results indicate that S100A9 is a potent stimulator of neutrophil trafficking to the lung, by enhancing Mac-1 expression and CXC chemokine generation in abdominal sepsis. Blocking S100A9 reduces neutrophil infiltration and activation and protects against edema formation and tissue damage in septic lungs. Thus, our study reveals an important role of S100A9 in septic lung damage and suggests that S100A9 could be a useful target to attenuate lung inflammation and tissue damage in abdominal sepsis.

## 4. Materials and Methods

### 4.1. Animals

All experimental procedures were performed according to suggestions from the Regional Ethics Committee for animal experimentation at Lund University, Sweden (Permit number: 5.8.18-08769/2019). Male C57BL/6 mice (weight, 20–25 g) were housed under standardized 12 h light–dark cycle conditions at 22 °C, fed a laboratory diet, and had water accessible ad libitum. Animals were anesthetized by intraperitoneal (i.p.) injection of 75 mg/kg of ketamine hydrochloride (Hoffman-La Roche, Basel, Switzerland) and 25 mg/kg of xylazine (Janssen Pharmaceutica, Beerse, Belgium). Buprenophin hydrochloride (0.5 mg/kg; Schering-Plough, Berkeley Heights, NJ, USA) was subcutaneously injected for analgesia. Mice were randomly assigned to different groups in all experiments.

### 4.2. Induction of CLP and Experimental Design

Induction of polymicrobial sepsis was performed as described previously [3]. For preparation, a midline incision was made to expose the cecum on the anesthetized mice. Subsequently, 75% of the cecum was ligated with a suture and soaked with phosphate-buffered saline (PBS; PH 7.4), and then was punctured twice with a 21-gauge needle. A small amount of feces from the perforation sites was gently squeezed out. The cecum was then returned to the abdominal cavity. Finally, the incision on the peritoneum was closed with 5-0 suture. In the sham group, the cecum was exposed but no ligation or puncture was performed. In experiments involving anti-S100A9 treatment, mice were randomly chosen and pre-treated with intraperitoneal (i.p.) injections of vehicle or a specific S100A9 blocker, ABR-238901 (10 mg/kg or 30 mg/kg, a gift from Active Biotech, Lund, Sweden), 1 h before induction of CLP or Sham. In separate experiments, ABR-238901 was given 4 h after CLP induction. Mice were re-anesthetized for sample collection. Blood collection, lung harvesting, and bronchoalveolar lavage fluid (BALF) collection were performed 6 h or 24 h after CLP induction.

### 4.3. Myeloperoxidase Activity

Lung tissues were pre-weighed and homogenized in 1 mL of phosphate-buffered saline (PBS) by a TissueLyser (Qiagen, Hilden, Germany). MPO activity of the supernatants were determined spectrophotometrically as the MPO catalyzed the change in absorbance in the redox reaction of H2O2 (450 nm, with a reference filter 540 nm, 25 °C). Results are interpreted as MPO units per gram of tissue.

### 4.4. Enzyme-Linked Immunosorbent Assay

Lung tissues were homogenized by a TissueLyser. Blood was collected from the inferior vena cava with acid citrate dextrose, centrifuged 2000× *g* for 10 min at room temperature, and the plasma was collected. Plasma and lung levels of S1009A were detected using Mouse S100A9 ELISA Kit (Abcam, Amsterdam, The Netherlands). Levels of CXCL-1, CXCL-2 and IL-6 in the lung were determined in supernatants by use of ELISA kits (R&D Systems Europe, Abingdon, Oxon, UK) according to the manufacturers’ instructions.

### 4.5. Histology

Samples of the lung were fixed in 4% formaldehyde for 24–48 h at 4 °C, then dehydrated with ethanol. Tissues were embedded in paraffin and 5 μm-thick sections were sliced and subsequently stained with hematoxylin and eosin. Histological evaluation was performed in a double-blinded manner based on a pre-established scoring system, as described previously (5). Briefly, four parameters were assessed, including size of alveolar spaces, thickness of alveolar septa, alveolar haemorrhage, and neutrophil infiltration. Each parameter was scored from 0 (absent) to 4 (extensive).

### 4.6. Bronchoalveolar Lavage Fluid (BALF) and Lung Edema

Animals were anesthetized and the trachea was exposed, a transparent PE50 catheter was placed into the trachea and fixed with suture. BALF was collected by washing five times with 0.8 mL cold PBS containing 1% EDTA. For counting leukocytes in BALF, BALF was centrifuged 1400 rpm for 5 min and the supernatant was discarded, and the pellet was resuspended in 200 μL PBS. Leukocytes were classified as monomorphonuclear (MNL) and polymorphonuclear (PMNL) leukocytes and counted in a Burker chamber. The left lung was excised, all other extrapulmonary tissues were removed and gently dried with blotting paper, and wet weight was measured. Samples from the lung of each animal were placed in a dish and dried at 60 °C for 3 days and weighed again at the dry weight. Lung edema was interpreted as a wet-to-dry weight (wet/dry) ratio.

### 4.7. Systemic Leukocytes Differential Counts

Blood was collected from a tail vein and directly mixed with Turks solution (1:20 dilution). Leukocytes were classified as monomorphonuclear (MNL) and polymorphonuclear (PMNL) leukocytes and counted in a Burker chamber.

### 4.8. Flow Cytometry

For analysis of Mac-1 expression, freshly isolated bone marrow neutrophils were fixed with 4% formaldehyde and then washed twice with PBS containing 2% FBS. To reduce nonspecific binding, FcγRIII/FcγRII was blocked with anti-CD16/CD32 (553124, BD Bioscience, San Diego, CA, USA), and continued labeling with FITC-conjugated anti-CD11b (553310, BD Biosciences, San Diego, CA, USA) and APC-conjugated anti-Ly6G (127614, Biolegend, London, UK). Flow cytometric detection was performed using the CytoFLEX Flow Cytometer (Beckman Coulter, Indianapolis, IN, USA) and data was analyzed by CytExpert 2.0 software (Beckman Coulter, Indianapolis, IN, USA).

### 4.9. Statistics

Graphpad Prism 8 was used for data analysis. Data are presented as box plots with median (25–75 percentile) and the whiskers extend from the minimum to the maximum levels and dots represent sample values. Statistical comparisons were performed using non-parametrical tests (Mann–Whitney or Kruskal–Wallis on ranks followed by Dunn’s multiple comparisons). *p* < 0.05 was considered significant and n represents the number of animals or experiments.

## Figures and Tables

**Table 1:** Systemic leukocyte counts.

	PMNL	MNL	Total Leukocytes
Sham	1.2 ± 0.2	5.3 ± 1.9	6.5 ± 1.8
Vehicle + CLP	0.6 ± 0.3 ^#^	2.0 ± 0.8 ^#^	2.6 ± 1.1 ^#^
ABR + CLP	1.3 ± 0.5 *	3.2 ± 0.7	4.6 ± 0.9 *

Mice were pre-treated with intraperitoneal injections of the vehicle or ABR-238901 (10 mg/kg) 1 h before Sham or CLP operation. Blood was collected from animals 24 h after CLP induction and leukocytes were identified as polymorphonuclear leukocytes (PMNL) and monomorphonuclear (MNL). Data are presented with mean ± SEM, 10^6^ cells/mL. *^#^ p* < 0.05 vs. Sham, * p < 0.05 vs. Vehicle, *n* = 5.

## Data Availability

Not applicable.

## Supplementary Materials

The following are available online at https://www.mdpi.com/article/10.3390/ijms222312923/s1.
