# Supplementary material for: Targeting S100A9 Reduces Neutrophil Recruitment, Inflammation and Lung Damage in Abdominal Sepsis
Source: Int J Mol Sci. 2021 Nov 29;22(23):12923. doi: 10.3390/ijms222312923 (PMC8658007; doi:10.3390/ijms222312923)
Supplement: Supplementary file 1 [file ijms-22-12923-s001.zip › ijms-1440377-supplementary.pdf]

S100A9 Mediates Neutrophil Recruitment, Inflammation and Lung Damage in Abdominal Sepsis

Zhiyi Ding [1], Feifei Du [1], Richard Garland Averitt V [1], Gabriel Jakobsson[2], Carl-Fredrik Rönnow [1], Milladur Rah-man [1], Alexandru Schiopu [2] and Henrik Thorlacius [1,*]

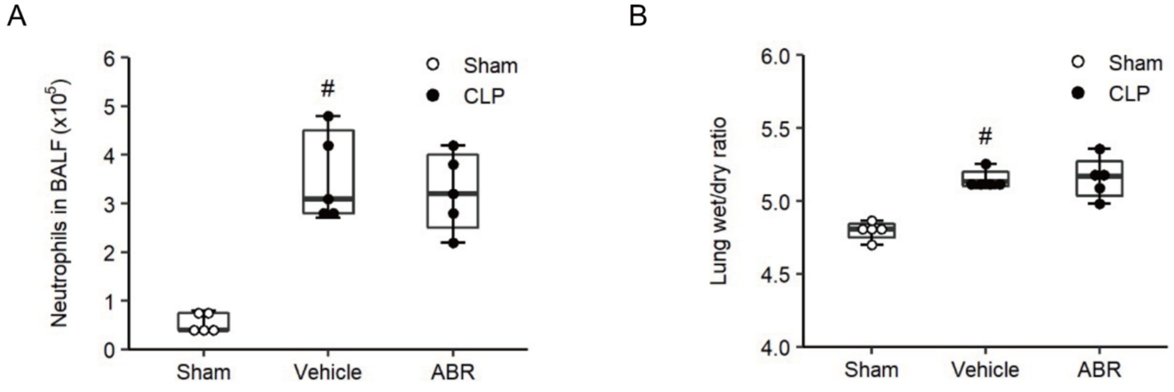

Supplementary Figure S1. Mice were treated with intraperitoneal injections of the vehicle or ABR (10 mg/kg) at 4h post CLP. (A) BALF neutrophils were quantified in bronchoalveolar lavage fluid collected 24h after CLP induction. (B) Lung were collected 24h after CLP and lung weight Wet/dry ratio was used to determine edema formation. Data are presented as box plots with median (25-75 percentile) and the whiskers extend from the minimum to the maximum levels and dots represent sample values, $^{\#}P < 0.05$ vs. Sham, and $^{*}P < 0.05$ vs. Vehicle, n = 5.

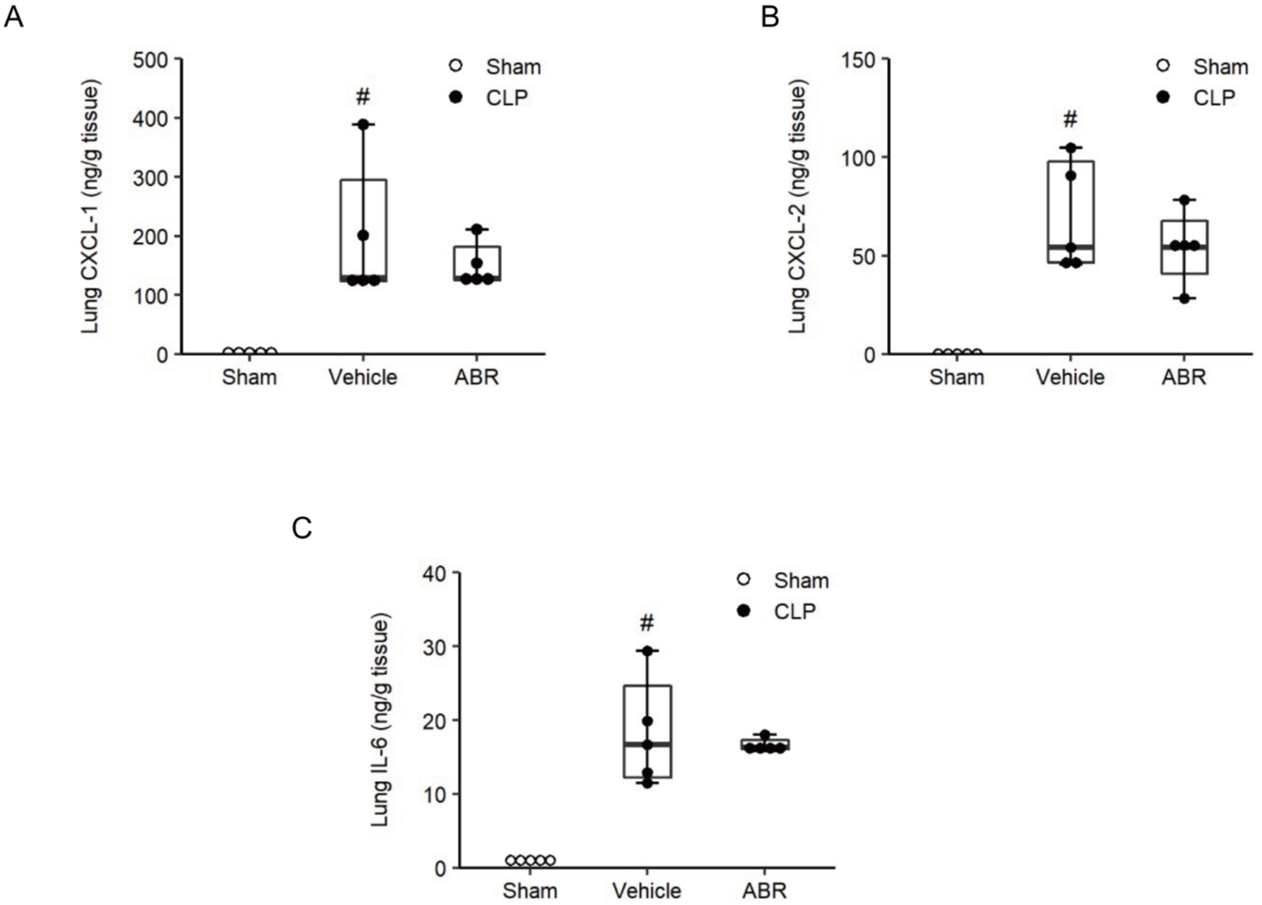

Supplementary Figure S2. Mice were treated with intraperitoneal injections of the vehicle or ABR (10 mg/kg) at 4h post CLP. Levels of (A) CXCL-1, (B) CXCL-2 and levels of (C) IL-6 in the lung were determined 24h after CLP induction. Data are presented as box plots with median (25-75 percentile) and the whiskers extend from the minimum to the maximum levels and dots represent sample values, $^{\#}P < 0.05$ vs. Sham, and $^{*}P < 0.05$ vs. Vehicle, n = 5.
